# Wessex Branch of the Association of Clinical Pathologists

**Published:** 1986-04

**Authors:** 


### Wessex Branch of the Association of Clinical Pathologists

The Autumn Meeting was held at the Edward Jenner Teaching Centre of the Royal Infirmary, Bristol, on 19 November. It was conducted under the Presidency of Dr. B. Murphy (Torbay Hospital), with Dr. K. A. V. Cartwright (Public Health Laboratory Service, Gloucester) as the local Branch Secretary. The meeting was attended by approximately 50 trainee and consultant pathologists working in the Haematology, Chemical Pathology, Medical Microbiology and Histopathology laboratories of the South Western Region and Bath. The following papers, of which abstracts are printed below, were presented to the Association during the afternoon Scientific Session. The ACP Wessex Branch was most appreciative of the active support of the Edward Jenner Centre staff, and pharmaceutical sponsors, for their help in making the meeting possible.

### 'UNDIFFERENTIATED' CARCINOMAS OF THE COLON

S.Z. Al-Sam and J. D. Davies, Department of Pathology, Royal Infirmary, Bristol, BS2 8HW

We have studied the morphological and immunohistochemical characteristics of over 30 colonic carcinomas. Many of the cases were kindly provided by Professor N. M. Gibbs, working in Guildford, for immunohistochemical and morphological evaluation.

A small proportion of colonic carcinomas belie their apparently aggressive appearance. These 'undifferentiated' carcinomas differ in two important respects from poorly differentiated adenocarcinomas of the colon. First, their component cells are capable of phenotypic expression of the immune secretory transport system with cytoplasmic immunoreactivity for Secretory Component, IgA and J-chain. Second, such tumours do not behave as badly as do conventional poorly differentiated colonic carcinomas: their lethal capacity is much less than is found in poorly differentiated adenocarcinomas of the colon. On the basis of these differences, together with their cytoplasmic argyrophilic properties, we venture to suggest that these tumours differ both in histogenesis and clinical behaviour from the more common poorly differentiated adeno-carcinomas of colon.

In a practical sense, the morphological diagnosis of these 'undifferentiated' carcinomas is capable of support by their functional peculiarities. They merit distinction from the more aggressive, and common, poorly differentiated adenocarcinomas arising in the large bowel. These peculiar tumours seem to represent an unrecognised line of differentiation.

### A CASE OF PYOMYOSITIS

I. Muscat, Public Health Laboratory, Heavitree, Exeter EX2 5AD

A 66-year old Caucasian man, with a past history of gout and myocardial infarction developed increasingly severe generalised myalgia over 4 days. There was no history of preceding malaise, trauma or tropical travel. He was admitted in septicaemic shock and died within 12 hours, despite treatment. Blood cultures grew *Staphylococcus aureus*. At post-mortem examination abscesses typical of pyomyositis were found in skeletal muscle. Haematogenous abscesses were also found in the myocardium and lungs.

Pyomyositis can be defined as primary *Staph. aureus* intramuscular abscess formation. Review of the literature indicates that abscesses are single in 60% of cases and tend to occur in the larger muscles. The condition is common in the tropics, and patients usually present with fever and tender, swollen muscles and loss of their function. A fulminating presentation is also described. Blood cultures are positive in 5% of cases. Curiously, muscle enzymes are not raised. Suitable imaging techniques and aspiration of the lesions are usually required for early diagnosis in temperate countries. Drainage and antibiotics are curative in 98% of the cases on record.

Pyomyositis is thought to arise from a transient bacteraemia with seeding into damaged muscle. There was no obvious cause of antecedent muscle damage in our case.

### NEOPLASTIC ANGIOENDOTHELIOSIS

G. S. Rutherfoord and D. Betty Brownell, Department of Neuropathology, Frenchay Hospital, Bristol

Neoplastic angioendotheliosis is a rare disorder characterised by either a focal or, more frequently, systemic intravascular proliferation of malignant cells which usually presents clinically as a vascular occlusive syndrome. In many instances the disease process primarily involves the cerebral vasculature leading to clinically confusing symptoms and signs that often defy accurate diagnosis. The neoplastic cells appear to be of endothelial origin, although evidence in support of this hypothesis is rather tenuous. Its basis in occasional case reports rests upon the authors describing the presence of Weibel-Palade bodies. Our paper presents a further two cases of this condition. In each instance a full autopsy failed to reveal any primary, focal malignancy and in both cases only *intravascular* tumour cells stained positively for Factor VIII-related antigen. However, both intravascular *and* extravascular tumour cells showed immunostaining with antisera to Leucocyte Common Antigen by means of the peroxidase–antiperoxidase technique. The significance of these results were discussed.

### SPURIOUS SMOOTH-MUSCLE TUMOURS IN RECTAL BIOPSIES: A WARNING

E. K. Dankwa, Judith L. Channer and J. D. Davies, University Department of Pathology, Royal Infirmary, Bristol, BS2 8HW

Smooth-muscle tumours affecting the muscularis mucosae of the rectum and sigmoid colon are excessively rare. Lesions simulating such tumours are, we believe, much more common. Having reviewed the rectal biopsies of the past 10 years in the Bristol Royal Infirmary we wish to point out the potential diagnostic errors in this field.

We think that any clinician should beware of an histological diagnosis of a 'muscular hamartoma' or 'leiomyoma' of the rectum or sigmoid colon resulting from a colonoscopically normal mucosa. Compression of muscular tissues may produce a deceptive appearance in the biopsy material, which may result in an erroneous histopathological diagnosis.

Histological reappraisal of a consecutive series of over 500 rectal biopsies has shown this potential misdiagnosis in approximately 2% of rectal biopsies. Awareness of the possible artefactual nature of these lesions is necessary in order to obviate quite needless clinico-pathological deliberation. Histologically the muscular lesions are centrally situated in the individual rectal biopsies, they are more frequent in 'twisted' biopsies and their appearances suggest that they are derived from artefactual dislocation of muscle from the muscularis mucosae into the lamina propria or from the muscularis propria to the muscularis mucosae. Hitherto such artefactual lesions do not appear to have been reported in the gastrointestinal or pathological literature. We suspect they may be misinterpreted as tumours or malformations.

### TESTING OF COAGULASE NEGATIVE STAPHYLOCCI FOR INTRINSIC RESISTANCE TO ß-LACTAM ANTIMICROBIALS BY DISC-DIFFUSION METHOD

**E. P. Moore and D. F. J. Brown,**
**Department of Microbiology, Royal Infirmary, Bristol, BS2 8HW**

An investigation was carried out to determine whether some of the methods found to be suitable for testing the susceptibility of *Staphylococcus aureus* to penicillinase resistant β-lactam antimicrobials in routine diagnostic laboratories were also satisfactory for the testing of coagulase–negative staphylococci. Ninety-three isolates of coagulase–negative staphylococci judged to be of clinical significance were tested by disc-diffusion using a variety of media, additives and incubation conditions against three β-lactam antimicrobials. Fifty-seven of the organisms demonstrated resistance under one or more test conditions. The results emphasise the heterogeneity of this group of organisms with a variety of different patterns of resistance being seen.

The organisms did not appear to express their resistance as reliably as *S. aureus* under conditions such as incubation at 30°C, or in the presence of 5% sodium chloride. This was especially so with Isosensitest agar, where incubation at 30°C, and more particularly the presence of sodium chloride commonly inhibited the expression of resistance that was obvious under other conditions. Resistance was demonstrated better on Mueller Hinton agar than with Isosensitest agar and, in contrast to the effect of its addition to Isosensitest agar, the addition of 5% sodium chloride to Mueller Hinton agar made the resistance of many strains more obvious. Methicillin (5 μg disc), Oxacillin (5 μg disc) and Cephradine (30 μg disc), were compared. Methicillin appeared to be the best agent for eliciting resistance to oxacillin or cephradine. Under most conditions a 48 hours incubation was necessary for optimal detection of resistance, although with Meuller Hinton agar containing sodium chloride, incubation at 37°C for 24 hours gave a good indication of resistance.

### THE ASPIRIN DOSE PROBLEM IS NOW SOLVED

**R. D. Eastham, Department of Clinical Pathology,**
**Frenchay Hospital, Bristol**

Recent work has shown that 80 mg per day of low-dose slow-release aspirin results in 95% inhibition of platelet cyclo-oxygenase by 4–7 days, with reduced thromboxane A activity (leading to platelet aggregation) and increased platelet sensitivity to PGI and PGE (anti-aggregation activity), without gastric irritation. Low-dose aspirin is compatible with both dipyridamol and warfarin therapy, and would therefore be useful in rheumatic heart valve disease, amaurosis fugax, the prevention of myocardial infarction, post-myocardial infarctional problems, and in post-coronary artery bypass complications, etc.

People wishing to indulge in self-help in the prevention of cardiovascular disease by the correction and avoidance of obesity, the correction of hypertension, the taking of a high-fibre diet, not smoking (primary and secondary) and, if one must, jogging, could undertake a series of simple measures which favourably affect multifactorial coronary heart disease. Low-dose slow-release aspirin (in the form of 20 mg q.d.s.) will inhibit platelet cyclo-oxygenase activity, without affecting vessel-wall prostacyclin production and raises plasma HDL, which is inversely associated with CHD mortality. Additionally the unsaturated fatty acids from two fatty fish meals taken weekly may also help reduce the incidence of death from coronary heart disease.

### THE ACUTE–PHASE RESPONSE

**C. E. Spence, Department of Chemical Pathology,**
**Royal Infirmary, Bristol, BS2 8HW**

Tissue injury results in an increase in the concentration of certain plasma proteins, mostly liver-derived, involved in the inflammatory response. Their synthesis is induced by interleukin 1 secreted by macrophages. Interleukin 1 also causes the associated fever, leucocytosis and muscle proteolysis in tissue injury.

The acute–phase response facilitates phagocytosis, the removal of damaged tissue and enables repair to occur. The functions of the proteins form a complex network also involving self-limiting control. The response can be rather crudely assessed by measuring the ESR, but in nearly all clinical applications the specific measurements of C-reactive protein yields the most useful information. Other acute-phase proteins are serum amyloid A protein, orosomucoid, α1-antitrypsin and haptoglobin. C-reactive protein has a short response time, may show up to a 100-fold increase in plasma level at the peak of the acute-phase response and has a short half-life. Its assay helps in the detection of certain organic diseases; for example it may distinguish inflammatory bowel disease from irritable bowel syndrome. It is also helpful in the assessment of the activity and prognosis of rheumatoid arthritis, its monitoring and treatment, and in detecting possible intercurrent infection in patients with leukaemia.

There is an interesting observation, under current investigation, that the acute-phase response seems to be deficient or becomes altered in certain inflammatory conditions.

### NECROTIC LYMPH NODES AND THEIR CONNECTIVE-TISSUE FRAMEWORK

**Judith L. Channer and J. D. Davies,**
**University Department of Pathology, Royal Infirmary, Bristol, BS2 8HW**

There is a wide range of types of necrosis to be found in lymph nodes. These include tuberculosis, cat-scratch disease, necrotic primary lymphomas, carcinomatous metastases, and 'spontaneous' lymph-node infarcation. The morphological interpretation of such lesions may, alas, lead to erroneous diagnosis.

Examination of over 60 blocks of the necrosis in lymph-node lesions by means of haematoxylin-and-eosin stains together with Gordon & Sweets' reticulin, trichrome and elastic-van Gieson preparations appeared to facilitate the correct diagnosis of the underlying nature of the lymph-node necroses. These conjoint procedures are more useful than the traditional histopathological stains aimed to demonstrate micro-organisms, which today are often lacking in necrotic lymph-node lesions of infective aetiology.

The well established connective-tissue stains that were used are capable of indicating the pre-existing structure of otherwise apparently amorphous necrotic lymph node lesions. In this way it was possible to diagnose in 90% of cases both the existence of primary and secondary tumours, and to differentiate these from infective or banal ischaemic necrosis. We believe that the use of several connective-tissue stains is of immediate practical value in establishing the cause of necrosis in lymph-node lesions. The omission of their use appears to be a false economy, which may result in erroneous or delayed diagnosis. Such potentially avoidable errors appear to have little justification, even in current economic circumstances. An unanticipated result of our morphological investigations was the detection of two types of necrosis in tuberculous lymphadenitis. As a consequence we believe that caseous necrosis is a two-stage sequence. Later, massive, necrosis may well have a different pathogenesis.

